# Eating Disorders Intensive Treatment (EDIT) Subteam: Shoring Up MDT Working to Turn the Tide for Patients at Risk of Hospitalisation

**DOI:** 10.1192/bjo.2024.444

**Published:** 2024-08-01

**Authors:** Alyssa Tan Jingren, Katherine Morton, Kelly Nugent, Nyree Weir

**Affiliations:** 1 Tertiary Eating Disorders Specialist Service, Lanarkshire, United Kingdom; 2 Burnbank Medical Practice, Hamilton, United Kingdom

## Abstract

**Aims:**

Presentations of severe Eating Disorders (ED) to the Tertiary Eating Disorders Specialist Service (TESS) in Lanarkshire have increased in recent years. Our criteria has also expanded to include severe Avoidant-Restrictive Food Intake Disorder (ARFID), increasing demand for a multidisciplinary team (MDT) approach for patients at high physical risk with less typical ED presentations. Medical Emergencies in Eating Disorders (MEED) recommends MDT working and development of pathways to support these patients.

The “EDIT subteam” was thus developed in March 2023, comprising: TESS psychiatrist, TESS GP, dietician, assistant practitioner, and TESS psychologist.

For TESS patients at high physical risk, high risk of hospitalisation, and who would benefit from a trial of “stepping up” treatment, we aimed to employ coordinated MDT intervention to 1. optimise community treatment, 2. regularly review risk and 3. reduce need for hospital admission.

**Methods:**

Each patient was discussed at a weekly MDT meeting attended by EDIT subteam, where risk assessment and management plan was agreed.

6-month review was conducted using meeting minutes, staff survey and group discussion, with consideration given to: number of patients prevented from requiring hospital, number of patients admitted to hospital and consideration if different levels of intervention could have prevented this, staff satisfaction and review of the MDT complement.

**Results:**

22 patients – 17 female, 5 male – were included on EDIT for the first 6 months. At point of step-down from EDIT, 13 had ongoing TESS community input, 5 were admitted to hospital, 3 were discharged from TESS and 1 transferred to Community Mental Health Team.

Most EDIT patients received input from multiple domains of the MDT. Given baseline low admission rates and complexity of patient presentation, we were unable to determine how many hospital admissions were prevented, but consensus was that overall, a higher level of care was provided. It was not felt that different levels of intervention could have prevented any of the 5 admissions. Staff feedback was positive: EDIT improved communication, provided job role diversification, contained and shared risk, improved awareness of care plans and resulted in better-considered onward referrals.

Areas for improvement included a lack of Occupational Therapy and nursing, and concern about EDIT patients skipping waiting lists.

**Conclusion:**

The EDIT subteam provides an avenue for high risk patients to be regularly discussed in an MDT setting – although impossible to empirically quantify if admissions were reduced, consensus within TESS was that the introduction of EDIT has improved community treatment for this group of patients.

